# 500-year climate cycles stacking of recent centennial warming documented in an East Asian pollen record

**DOI:** 10.1038/srep03611

**Published:** 2014-01-09

**Authors:** Deke Xu, Houyuan Lu, Guoqiang Chu, Naiqin Wu, Caiming Shen, Can Wang, Limi Mao

**Affiliations:** 1 Key Laboratory of Cenozoic Geology and Environment, Institute of Geology and Geophysics, Chinese Academy of Sciences, Beijing 100029, China; 2 Key Laboratory of Plateau Lake Ecology and Global Change, Yunnan Normal University, Kunming, Yunnan 650092, China; 3 University of Chinese Academy of Sciences, Beijing 100049, China; 4 State Key Laboratory of Paleobiology and Stratigraphy, Nanjing Institute of Geology and Paleontology, Chinese Academy of Sciences, Nanjing 210008, China

## Abstract

Here we presented a high-resolution 5350-year pollen record from a maar annually laminated lake in East Asia (EA). Pollen record reflected the dynamics of vertical vegetation zones and temperature change. Spectral analysis on pollen percentages/concentrations of *Pinus* and *Quercus*, and a temperature proxy, revealed ~500-year quasi-periodic cold-warm fluctuations during the past 5350 years. This ~500-year cyclic climate change occurred in EA during the mid-late Holocene and even the last 150 years dominated by anthropogenic forcing. It was almost in phase with a ~500-year periodic change in solar activity and Greenland temperature change, suggesting that ~500-year small variations in solar output played a prominent role in the mid-late Holocene climate dynamics in EA, linked to high latitude climate system. Its last warm phase might terminate in the next several decades to enter another ~250-year cool phase, and thus this future centennial cyclic temperature minimum could partially slow down man-made global warming.

Periodic climate changes on multidecadal to multicentennial scales induced by external and internal forcings are of importance not only for understanding the regional climate dynamic but also for assessing the contribution of anthropogenic forcing to the 20th century warming^1^,^2^,^3^,^4^. Among these cyclic climate changes, the effect of the multicentennial scale solar variability is less certain. A ~500-year cycle in solar activity, inferred from global atmospheric ^14^CO_2_ production variation^5^, might have driven North Atlantic, North Pacific and North America terrestrial climates^6^,^7^,^8^,^9^,^10^. However, it remains to be seen whether this periodic paleoclimate changes is global or regional signals in the Holocene.

Observations and model simulations over recent centuries suggested that multidecadal to multicentennial cyclic change of solar activity and climate patterns (such as Pacific Decadal Oscillation, Atlantic Multidecadal Oscillation, and North Atlantic Deep Water) still existed under anthropogenic forcing and played an important role in regulating global to regional terrestrial (EA, North America, and Europe) and marine (North Atlantic and Pacific) climate change during the past centuries^9^,^10^,^11^,^12^,^13^,^14^,^15^. But recent studies based on global and regional climate records indicated that the warming of the past century is unprecedented and not relative to natural forcing but anthropogenic forcing^4^,^16^,^17^. One of reasons for this controversy rising is that multicentennial periodic variability in regional climate changes in other places of the world, especially in EA, is still not well known due to the lack of long-term and high-resolution regional climate proxies. Although work to date some hints of multidecadal to multicentennial-scale climate changes in EA have been revealed by high-resolution stalagmite^18^,^19^ and varved maar lake records^20^, there has been no detailed study of ~500-year periodic climate variability to the extent that we know either the patterns or mechanisms.

Here we presented a high-resolution pollen record from annually laminated maar Lake Xiaolongwan in EA to reconstruct the dynamics of regional vegetation and climate change during the past 5350 years. We further conducted spectra and wavelet analysis on temperature proxies reconstructed from pollen record to reveal its multicentennial oscillation. Finally, we discussed the mechanisms for this multicentennial climate oscillation.

## Results

### Site descriptions and material

**Figure 1:**
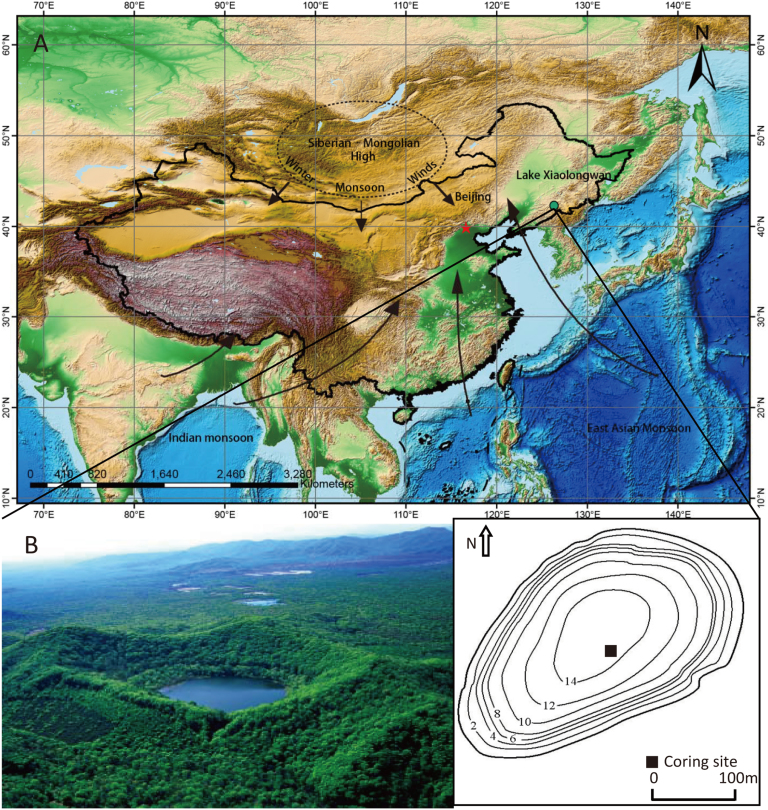
Overview maps of Lake Xiaolongwan showing locations of coring site. (A) Position, Green dot: Lake Xiaolongwan, Red dot: Beijing City. Regional atmospheric circulation (Black Arrows): Winter monsoon, East Asian Monsoon and Indian Monsoon. (B) A photo and a bathymetric curve sketch map of Lake Xiaolongwan. The firgure1A was generated using DIVA-GIS 7.5 (http://www.diva-gis.org/). The photo in Firgure 1B was taken by Guoqiang Chu.

Lake Xiaolongwan [42°18.0′N, 126°21.5′E, altitude: 655 m asl (above sea level)] is located in Longwan National forest park at the northwest margin of Changbai Mountain chain of Northeast China (Fig. 1). The highest peak of Changbai mountain on the Chinese side is 2691 m asl. The climate conditions in the study region are controlled by East Asian monsoon system. Summer monsoon from mid-low latitude ocean brings warm and humid air mass to this region in summer, whereas winter monsoon controlled by Siberia High causes cold and dry climate in winter^21^,^22^.

The regional vegetation in this mountain area is vertically zoned following the temperature gradient (Table S1). Broadleaved deciduous forest consisting of constructive species deciduous *Quercus mongolica* occupies the warm zone below 720 m asl with mean annual temperature (MAT) above 4°C. The mixed deciduous and coniferous forest, including *Pinus koraiensis*, *Abies holophylla*, *Carpinus cordota*, *Ulmus propinqua*, *Acer mono*, deciduous *Quercus mongolica*, *Juglans mandshurica*, *Betula costata, Betula platyphylla*, *Tilia amurensis*, and *Fraxinus mandshurica*, develops in this relative temperate zone from 720 to 1100 m asl with MAT of 3°C–4°C. In the cool zone with MAT of 0°C–2°C from 1100 to 1800 m asl occurs the coniferous forest dominated by *Pinus koraiensis*, *Picea koraiensis*, *Picea jazoensis*, and *Abies nephrolepis*. The coldest (MAT < 0°C) zone is occupied by subalpine *Betula ermanii* dwarf shrub from 1800 to 2100 m asl, and alpine tundra above 2100 m asl composed of *Dryas* *octopetala* and *Salix rotundifolia*^23^,^24^. The lake Xiaolongwan is surrounded by temperate mixed deciduous and coniferous forests. The forests are dominated by *Pinus koraiensis* and *Quercus mongolica*, together with some other broadleaved deciduous species such as *Carpinus cordota*, *Acer mono*, *Fraxinus mandshurica*, *Betula costata, Betula platyphylla*, *Juglans mandshurica*, and *Tilia amurensis*^25^.

### Chronology

A 271-cm piston core was raised from a site at water depth of 14.5 m near the center of the lake in the early spring of 2006 (Fig. 1). The varved sediments consist of grey- and brown- colored laminate couplets in the section^21^ (Fig. S1). The brown-colored layer, which is formed in autumn, is composed mainly of dinoflagellate cysts^21^. The grey-colored layer, which is formed in spring and summer, consists of other organic, siliceous matter (plant detritus, diatom, chrysophyte cysts) and clastics^21^. Previous studies using scanning electron and optical microscope, sediment trap observation, and independent chronology method indicated that the varved sediments were most likely annually laminated^21^. The upper 104 cm of annually laminated sediment covered 1600 years^20^,^26^. In this study, the varve counting result showed that the upper 271 cm of this core contained a maximum of 5350 layers.

**Figure 2:**
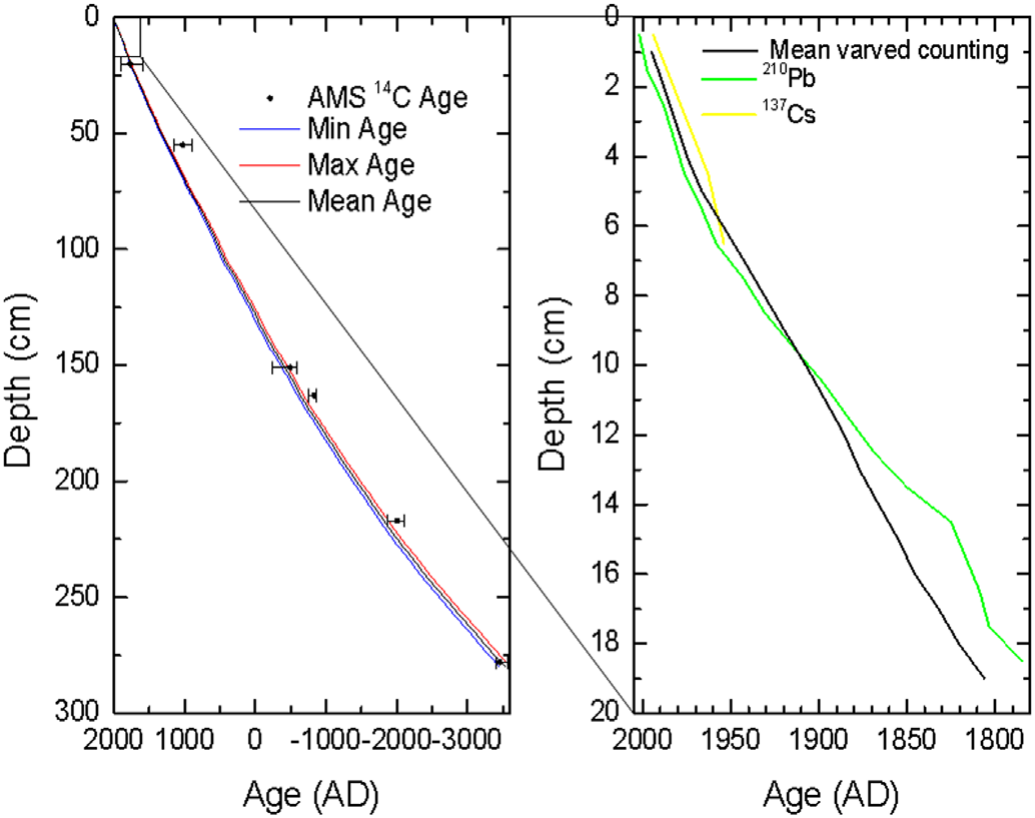
Comparative diagram of the sediment chronology derived from ^137^Cs, ^210^Pb, ^14^C and counting laminations of core Lake Xiaolongwan. Black dot: Calendar AMS ^14^C Age with dating errors, Blue line: Minimum varved age, Red line: Maximum varved age, Black line: Mean varved age, Green line: ^210^Pb age, Yellow line: ^137^Cs age.

Six radiocarbon ages were obtained from monospecific samples of bulk and leaf (Table S2). ^14^C ages were converted to calendar ages using CALIB 6.1 and IntCal09 data sets^27^. The age model of the core from varve counting was supported by independent chronological control using radio nuclides analyses including^137^Cs, ^210^Pb and ^14^C^26^ (Figs. 2 and S2). The results using the two dating methods indicated that the varved maximum age of this core covered the interval from 3340 BC to 2005 AD (Table S2 and Fig. 2).

### Pollen record indicated ~500-year cyclic vegetation and climate change

**Figure 3:**
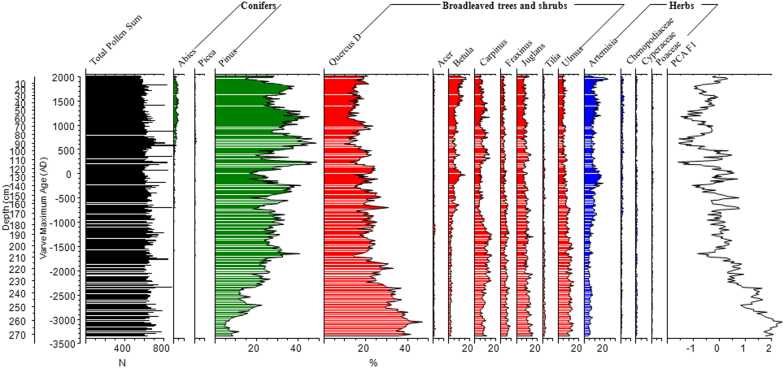
Pollen diagram of key pollen types from core Lake Xiaolongwan.

Pollen percentages of dominant pollen types, such as temperate mixed deciduous and coniferous forest elements [*Pinus*, *Betula*, *Carpinus*, deciduous *Quercus* (*Quercus* *D*) and *Ulmus*] and herb taxa (*Artemisia*) showed significant changes from 3340 BC to 2005 AD (Figs. 3 and S3). Among them, *Pinus, Betula,* and *Artemisia* exhibited an increase trend*,* while *Quercus D*, *Carpinus* and *Ulmus* showed a decrease trend. Superimposed on these two trends were quasi-periodic changes in pollen percentages of those pollen types.

Principal components analysis (PCA) was applied to the terrestrial pollen percentage data to extract main gradient changes in vegetation. All pollen taxa with relative abundance >2% in at least two samples were used in data analyses. The first and second principal components (PCA F1 and PCA F2) have eigenvalues of 0.71 and 0.15, explaining 71% and 15% total variance of pollen data respectively (Fig. S4). Broadleaved taxa, *Quercus D,* *Ulmus*, *Juglans*, *Fraxinus*, and *Carpinus* have positive loadings on axis 1, whereas temperate mixed deciduous and coniferous forest taxa, *Pinus*, *Abies*, *Betula* and some herb taxa, such as *Artemisia* and Chenopodiaceae, have negative loadings. The PCA F1 loadings reflect the dynamics of vertical vegetation belt indicated by pollen record in the study region.

*Pinus* is the main component of temperate mixed deciduous and coniferous forest, whereas *Quercus D* is the constructive taxa of the broadleaved deciduous forest^23^,^24^. In addition, the mountains within 5 km of the Lake Xiaolongwan, which ranges from ~500 to ~1000 m asl, are covered by temperate broadleaved forest, temperate mixed deciduous and coniferous forest, and their ecotone. Thus, the anti-phase fluctuations between *Pinus* and *Quercus D* represent the ratio changes between coniferous and broadleaved plants in the forest community^28^,^29^ and dynamics of vertical vegetation belt in Changbai Mountain region^30^,^31^ (Fig. 3 and Table S1).

The precipitation varies from 700 to 1400 mm in Changbai Mountain region, which is a typical temperate-humid oceanic monsoon climate zone^23^,^24^. Studies on the factors controlling the distribution of mountain vegetation zones indicate that, this dynamic of vertical vegetation zone is controlled by temperature rather than precipitation in the humid regions such as our study region within a typical temperate-humid oceanic monsoon climate zone^32^,^33^,^34^. Furthermore, broadleaved trees favor a warmer environment, while temperate mixed deciduous and coniferous forests are able to bear cooler condition than broadleaved forest in the mountain region of Northeast China^35^,^36^. Therefore, a high abundance of *Pinus* and low PCA F1 scores suggest downward of temperate mixed deciduous and coniferous forest and thus cool climatic conditions, whereas a high proportion of *Quercus D* and high PCA F1 imply upward of temperate deciduous forest and thus a warm conditions.

The decreases of *Quercus D* component and PCA F1 (increases of *Pinus* component) indicated the cooling trend during the past 5350 years (Fig. 3). In addition, the most striking feature is a series of multicentennial oscillations among *Pinus*, *Quercus D* and PCA F1 during the cooling trend (Fig. 3). *Pinus* component reaches its higher value, while *Quercus D* ratio and PCA F1 value drop to a low level around 2700 BC, 2200 BC, 1600 BC, 1200 BC, 900 BC, 600 BC, 300 BC, 200 AD, 700 AD, 1200 AD and 1800 AD. Each PCA F1 and *Quercus D* ratio lower value (*Pinus* higher value) interval is about 500 year. The regular 11 anti-phase coupled fluctuations between *Pinus* and *Quercus D* existed throughout the past 5350 years. Additionally, their concentrations display similar change (Fig. S5). Furthermore, these oscillations seem rather regular during the past 5350 years.

**Figure 4:**
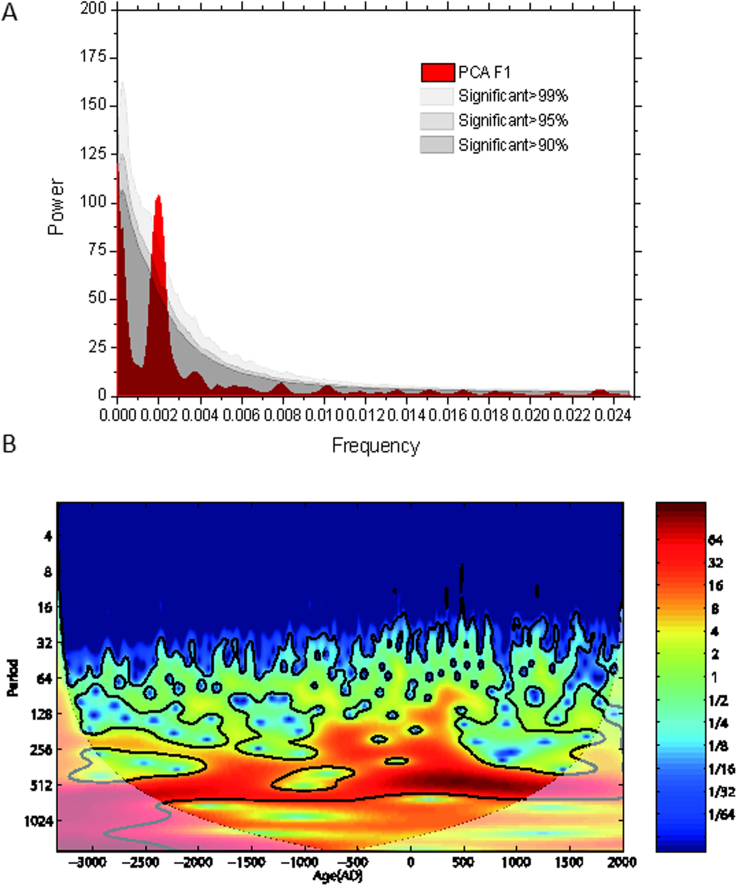
Characteristic PCA F1 periodicities. (A) Univariate spectral analysis results of the PCA F1 time series over the past 5350 years. The spectra were estimated using the Lomb-Scargle Fourier Transform for unevenly spaced data. OFAC and HIFAC is 2 and 1, respectively. It was performed by the Welch-Overlapped-Segment-Averaging procedure with 50% overlapped segments for univariate spectra. A Welch window type is employed to reduce spectral leakage. The univariate spectra were bias-corrected using 1000 Monte-Carlo simulation. The number within the graph indicates that significant periodicity of 500-year is above the 99% confidence level based on the χ^2^ test. The 6-dB bandwidth (BW), determining the frequency resolution, is 0.744 ky^−1^. See ref. 37 for details of the method. (B) Wavelet power spectrum of the PCA F1 records after interpolation to evenly spaced data. The shape of the mother wavelet was set to Morlet. High power is indicated by red whereas low power is indicated by blue. The regions enclosed by black line shows the confidence level greater than 95%. The dark shaded area indicates the cone-of-influence, where edge effects become important.

We performed spectral and wavelet analysis on PCA F1 and spectral analysis on the percentage and concentration of *Pinus* and *Quercus D* (Figs. 4 and S6) to determine their periods and periodic stabilities^37^,^38^. The spectra of PCA F1, *Pinus* and *Quercus D* reveal peaks at ~500-year period, significant at the 99% confidence level. The result of the wavelet analysis shows that 500-year quasi-periodic oscillations of PCA F1 are almost completely stable and dominant during the last 5350 years.

**Figure 5:**
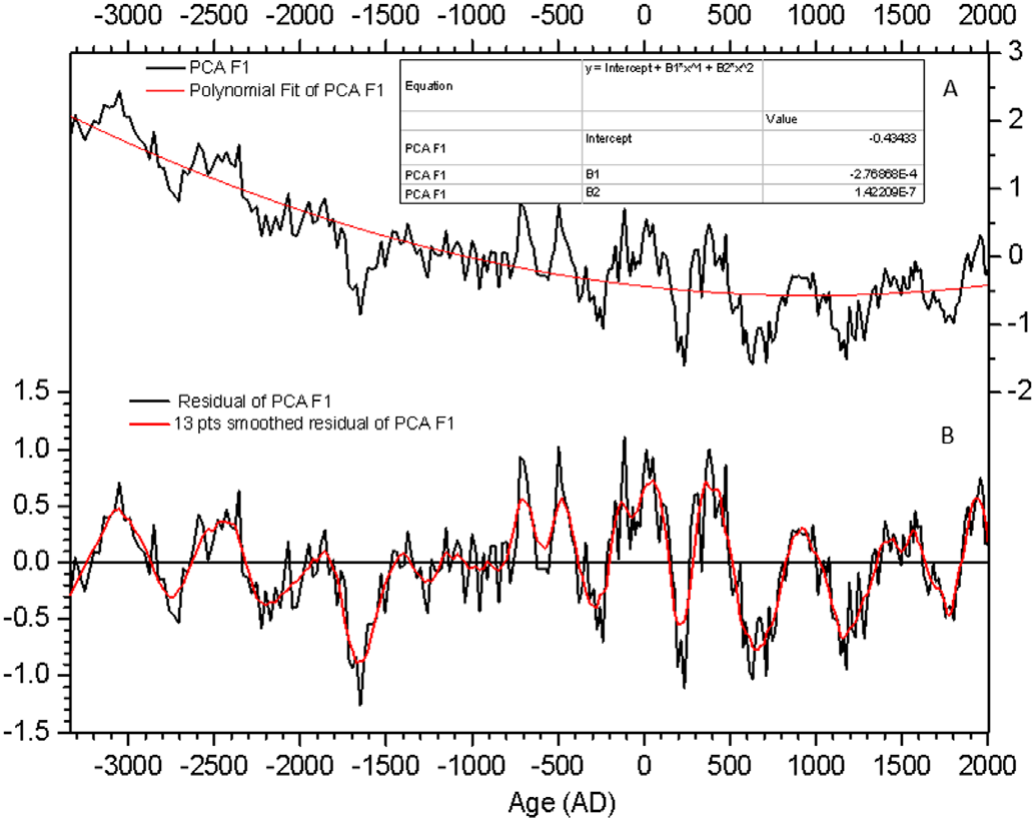
Time series of the PCA F1 record. (A) The PCA F1 data was detrended using polynomial fitting. (B) The original and 13 points smoothed residuals of PCA F1.

**Figure 6:**
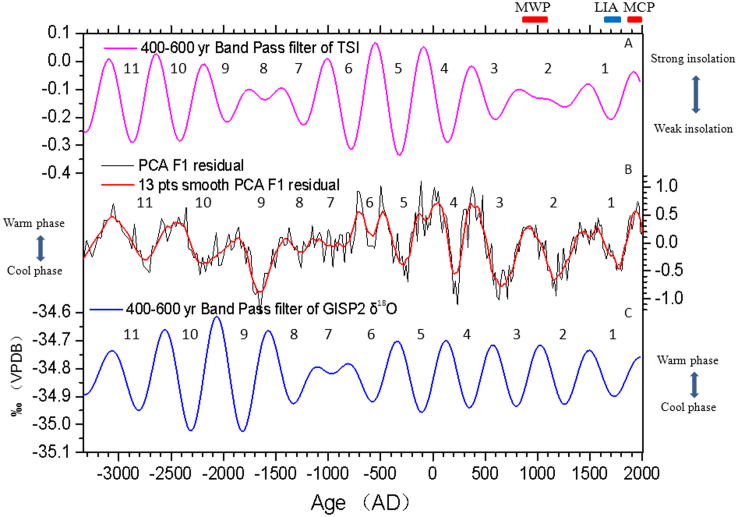
A comparison of climate proxy records from core Lake Xiaolongwan with other proxy records for Total Solar Irradiance and Greenland (GISP2) oxygen isotope (δ^18^O). (A) 400–600 year band pass filter of Total Solar Irradiance^5^,^39^, (B) Residuals of PCA F1, (C) 400–600 year band pass filter of GISP2 oxygen isotope (δ^18^O). VPDB, Vienna Peedee Belemnite. Two red bars and one blue bar show the timing of Medieval Warm Period (MWP), Modern Warm Period (MCP) and Little Ice Age (LIA), respectively.

PCA F1 loadings were detrended using polynomial fit (Figs. 5). The residuals show totally 11 ~ 500-year cool and warm oscillations. The ~500-year cycles are a few decades lag behind or almost in phase with 400–600 year-band-pass total solar irradiance (TSI)^5^,^39^ and Greenland (GISP2) oxygen isotope (δ^18^O)^8^ change (Fig. 6). Furthermore, high (low) *Pinus* and low (high) *Quercus* *D* components are correlated with each weak (strong) TSI period (Figs. S7 and S8). The lake records in North America and Pan-Arctic also showed ~500-year cyclic climate change^6^,^9^,^10^,^40^, suggesting that this phenomenon is widespread in the northern hemisphere continent.

During the last millennia, two warm (800 ~ 1100 AD and 1830s ~ 2005 AD) and one cold (1550 ~ 1830s AD) periods (Fig. 6) inferred from PCA F1 are almost in phase with the well-known Medieval Warm Period (MWP), Modern Warm Period (MCP) and Little Ice Age (LIA), respectively^41^. In the MCP, the abrupt rebound of PCA F1 is nearly synchronous with Greenland temperature^8^ and TSI^5^,^39^ changes, and this warming phase lasts nearly 170 years (Fig. 6). This recurrent warm phase would be ended within several decades according to the cyclic climate change in EA, if anthropogenic forcing were unconcerned. Thus, the coming of natural-forced ~250-year cooling phase could reduce the anthropogenic warming according to prediction of cyclic climate change in EA.

## Discussion

Comparison of late Holocene TSI, North Atlantic Deep Water (NADW) and EA climate indexes suggests that ~500-year periodic solar irradiance plays a key role in cyclic oceanic and atmospheric change^5^,^6^,^7^,^39^. Climate modeling and observation data provide a mechanism that solar forcing triggers the surface temperature variability and atmospheric dynamic^15^,^42^,^43^,^44^. If TSI reduces (increases), the downward-propagating effects triggered by changes in stratospheric ozone lead to cooling (warming) of stratosphere and global land surface temperature^15^,^42^,^43^.

Atmospheric responses to reduced solar irradiance could lead to the coincident increases in North Atlantic drift ice, reduces of NADW intensity, cooling of both the ocean surface and high-latitude continent around North Atlantic and North Pacific, and trigger the negative state of Arctic Oscillation/North Atlantic Oscillation (AO/NAO)^6^,^44^,^45^. When TSI reduced (increased), northern high-latitude terrestrial region experienced cool (warm) climate^5^,^6^,^15^,^45^. This effect could lead to the increases (decreases) drift ice and weaken (strengthen) the variability of NADW^5^,^6^,^45^.

In addition, AO/NAO and NADW can amplify EA land surface temperature change^6^,^44^,^46^,^47^. Because AO/NAO and NADW could regulate atmospheric transportation from northern hemisphere high-latitude region to mid-latitude EA^46^,^47^,^48^. In negative (positive) AO/NAO phase, EA continent could receive more (less) cold air from Siberian Mongolia High and be cooling down (warm up)^46^,^48^.

Thus, our study provides evidence for the influence of ~500-year solar variability on climate in EA. The solar variability associated with amplified AO/NAO and NADW anomaly could regulate land temperature variation over EA and trigger periodic vertical zonal vegetation change in Northeast Asia.

Our pollen record shows ~500-year cyclic vegetation and temperature oscillation in EA during the last five millennia. This result indicates that ~500-year cyclic climate change over East Asian still exists under anthropogenic forcing during the last century. Recent warm phase, which has lasted about 170 years, is most likely finished in several decades without regard to anthropogenic factors. In addition, this periodic climate change could reduce the man-made warming trend in the next two centennials in EA. Furthermore, this EA cyclic climate change may be influenced by solar irradiance induced AO/NAO and NADW anomaly. Our study indicates that EA climate system is highly sensitive to weak perturbations in the solar energy output on the multicentennial scale.

## Methods

The upper 19 cm of the core was sampled at 1-cm interval for ^137^Cs and ^210^Pb ages (Figs. 2 and S2). The sediment cores were sampled into slabs of 6.5 cm in length with a 1.5 cm overlap, then shock-frozen, vacuum-dried and impregnated with epoxy resin in advance of preparation for thin sections. Varves were identified and counted from thin sections at different magnifications (4×, 6.5×, 20×) using a Leitz optical microscope. The vacuum-dried samples of the cores were measured for radionuclides in a well-type germanium detector (EGPC 100P-15R). Each sample was packed in a 15 mm polyethylene tube and stored for 3 weeks in sealed containers to allow radioactive equilibration, was counted for 48 h. ^210^Pb was determined via its energy emission at 46.5 keV. ^226^Ra was determined by gamma spectrometry, based on the measurement of ^214^Pb (241.9, 295.2 and 351.9 keV) and ^214^Bi (609.3 keV) photo peaks. ^137^Cs was measured by its emissions at 662 keV. The unsupported activity ^210^Pb_unsup_ (subtracting ^226^Ra) was used to estimate average linear sedimentation rates for the core. Radiometric dates were calculated from the ^210^Pb and ^137^Cs records using the CRS model^49^.

Pollen samples of 0.3 ± 0.05 g at 1 cm intervals with average resolution of 20-yr from the same split cores were used. This interval was selected for thin section preparation for varve counting to ensure the best temporal correlation between the pollen and varve data. All samples were treated with KOH, HCl, HF and a hot acetolysis mixture. *Lycopodium* spores (27637 per tablet) were added to each sample to calculate pollen concentrations. Sample residues were in glycerine and analyzed by Leica DM750 microscope at ×400 magnification. Pollen preservation and concentration were great and an average sum of 652 (minimum 559, maximum 931) terrestrial pollen grains were counted for every sample (Fig. 3).

We used TILIA 1.7.16 computer programs^50^ to calculate and plot the pollen data. The pollen percentage diagram was constructed based on total terrestrial pollen, which excludes aquatic plants and ferns.

## Author Contributions

D.X. and H.L. designed research; G.C. organized field work with D.X. and H.L.; D.X., H.L., G.C. and N.W. performed research; D.X., H.L., G.C. and N.W. contributed new reagents/analytic tools; G.C. constructed the age-depth model; D.X., H.L., G.C., N.W. and C.S. analyzed data; G.C., D.X. and C.W. sampled and did core description. L.M. illustrated the pollen plate. D.X., H.L. and C.S. wrote the paper. All authors discussed the results and reviewed the manuscript.

## Supplementary Material

Supplementary Information SI manuscript
