# Supplementary material for: 500-year climate cycles stacking of recent centennial warming documented in an East Asian pollen record
Source: Sci Rep. 2014 Jan 9;4:3611. doi: 10.1038/srep03611 (PMC3885877; doi:10.1038/srep03611)
Supplement: Supplementary Information — SI manuscript [file srep03611-s1.doc]

Supplementary Information

**500-year climate cycles stacking of recent centennial warming documented in an East Asian pollen record**

Deke Xu^a1^, Houyuan Lu^a1^, Guoqiang Chu^a^, Naiqin Wu^a^, Caiming Shen^b^, Can Wang^a,c^, Limi Mao^d^

^a^Key Laboratory of Cenozoic Geology and Environment, Institute of Geology and Geophysics, Chinese Academy of Sciences, Beijing 100029, China

^b^Key Laboratory of Plateau Lake Ecology and Global Change, Yunnan Normal University, Kunming, Yunnan 650092, China

^c^University of Chinese Academy of Sciences, Beijing 100049, China

^d^State Key Laboratory of Paleobiology and Stratigraphy, Nanjing Institute of Geology and Paleontology, Chinese Academy of Sciences, Nanjing 210008, China

**This file includes:**

**Table S1**

**Table S2**

**Figure S1**

**Figure S2**

**Figure S3**

**Figure S4**

**Figure S5**

**Figure S6**

**Figure S7**

**Figure S8**

**Table S1 Vegetation distribution along the altitudinal gradient in Changbai mountain region**

| Altitude | Vegetation zone | Main taxa | Mean annual temperature |
| --- | --- | --- | --- |
| (masl) |  |  | °C |
| >2100 | Alpine tundra | *Dryas octopetala, Salix rotundifolia,* | <-4 |
| 1800-2100 | Sub-alpine dwarf shrub | *Betula ermanii* | -4-0 |
| 1100-1800 | Coniferous forest | ***Pinus koraiensis****, Picea jazoensis,* | 0-2 |
|  |  | *Picea koraiensis, Abies nephrolepis* |  |
| 720-1100 | Mixed deciduous and | ***Pinus koraiensis****, Abies holophylla,* | 3-4 |
|  | coniferous forest | *Carpinus cordota, Ulmus propinqua,* |  |
|  |  | *Acer mono,* ***Quercus mongolica****,* |  |
|  |  | *Fraxinus mandshurica, Juglans* |  |
|  |  | *mandshurica,Tilia amurensis,* |  |
|  |  | *Betula costata,Betula platyphylla* |  |
| <720 | Broadleaved deciduous forest | ***Quercus mongolica****,Corylus heterophylla* | >4 |
|  |  | *Ulmus propinqua,Tilia amurensis,* |  |
|  |  | *Juglans mandshurica, Acer mono* |  |
|  |  | *Populus ussuiensis* |  |

**Table S2 AMS ^14^C ages of core Lake Xiaolongwan**

| Lab No. | Depth | Dating materials | Radiocarbon Age | Corrected ^14^C age | Uncertainty | Calibrated Age | 2σ-range | |
| --- | --- | --- | --- | --- | --- | --- | --- | --- |
|  | (cm) |  | AD | AD | a | Cal AD | AD | |
| BA07706 | 20 | Bulk sediment | 1550 | 1762 | ±40 | 1772 | 1953 - 1645 | |
| BA07707 | 56 | Leaf | 735 | 947 | ±40 | 1028 | 1155 - 909 | |
| BA07708 | 154 | Leaf | -665 | -453 | ±40 | -489 | -393 - -748 | |
| BA07709 | 165 | Leaf | -930 | -718 | ±40 | -829 | -792 - -901 | |
| BA07710 | 219 | Leaf | -1900 | -1688 | ±40 | -2004 | -1898 - -2133 | |
| BA07711 | 278 | Leaf | -2890 | -2678 | ±35 | -3457 | -3348 - -3516 | |
| All the samples were done at the Radiocarbon Laboratory of Peking University, China. | | | | | | | |  |
| The reservoir correction factor is 212 years of the uppermost 20 cm of the core sediments. | | | | | | | |  |
| All Corrected ^14^C ages with 2σ-range were calibrated using the Intcal09 data set from the CALIB 6.1 program. | | | | | | | |  |

**Figure S1 Photomicrographs showing annual laminations in the sediments of Lake Xiaolongwan**


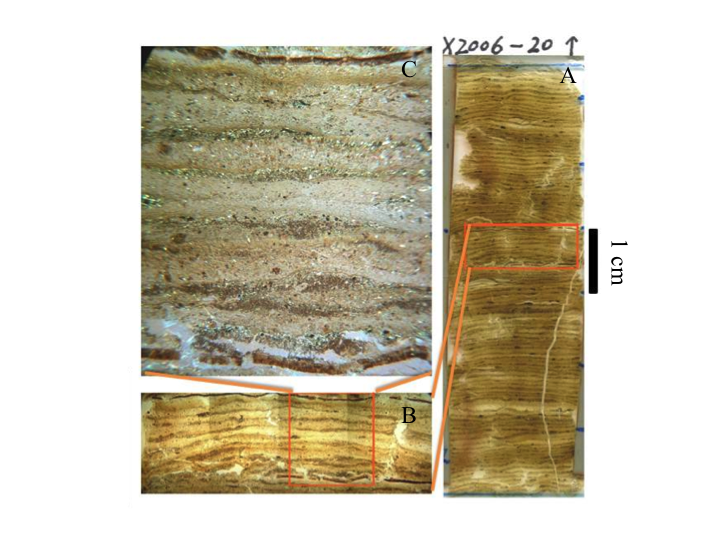


(**A**) Scanned from thin section X2006-20. (**B**) Detail of laminations from an oriange marked area in Figure S2A. (**C**) Detail of laminations from an oriange marked area in Figure S2B.

**Figure S2 The ^137^Cs, ^210^Pb and ^226^Ra vertical profiles for the sediment core Xiaolongwan.**

The ^137^Cs activity exhibits a sharp peak, suggesting that mixing processes of the sediments have not occurred. The highest ^137^Cs (41.1 dpm/g) occurred at a depth of 4.5 cm, and was deposited at or around the peak in above-ground nuclear weapons testing in 1963. The ^137^Cs value at a sediment depth of 6.5 cm is near zero (cesium horizon), and could have been deposited at or around the onset of large-scale nuclear weapons testing in the early 1950s. According to the cesium peak and horizon, average sediment accumulation rates in the core Lake Xiaolongwan are 0.11 and 0.12 cm/yr, respectively. The activities of ^210^Pb declined more or less exponentially with depth, indicating relatively uniform sediment accumulation during the past ~100 years except for an increase at 4.5 cm and a decrease at 3.5 cm. ^226^Ra activities are relatively uniform, with a mean value of 2.36 dpm/g. The ^210^Pb chronology was calculated using the CIC model[^3^](#_ENREF_3) to estimate sedimentation rates of the core Lake Xiaolongwan.

**Figure S3 A plate of common pollen types in the Lake Xiaolongwan**


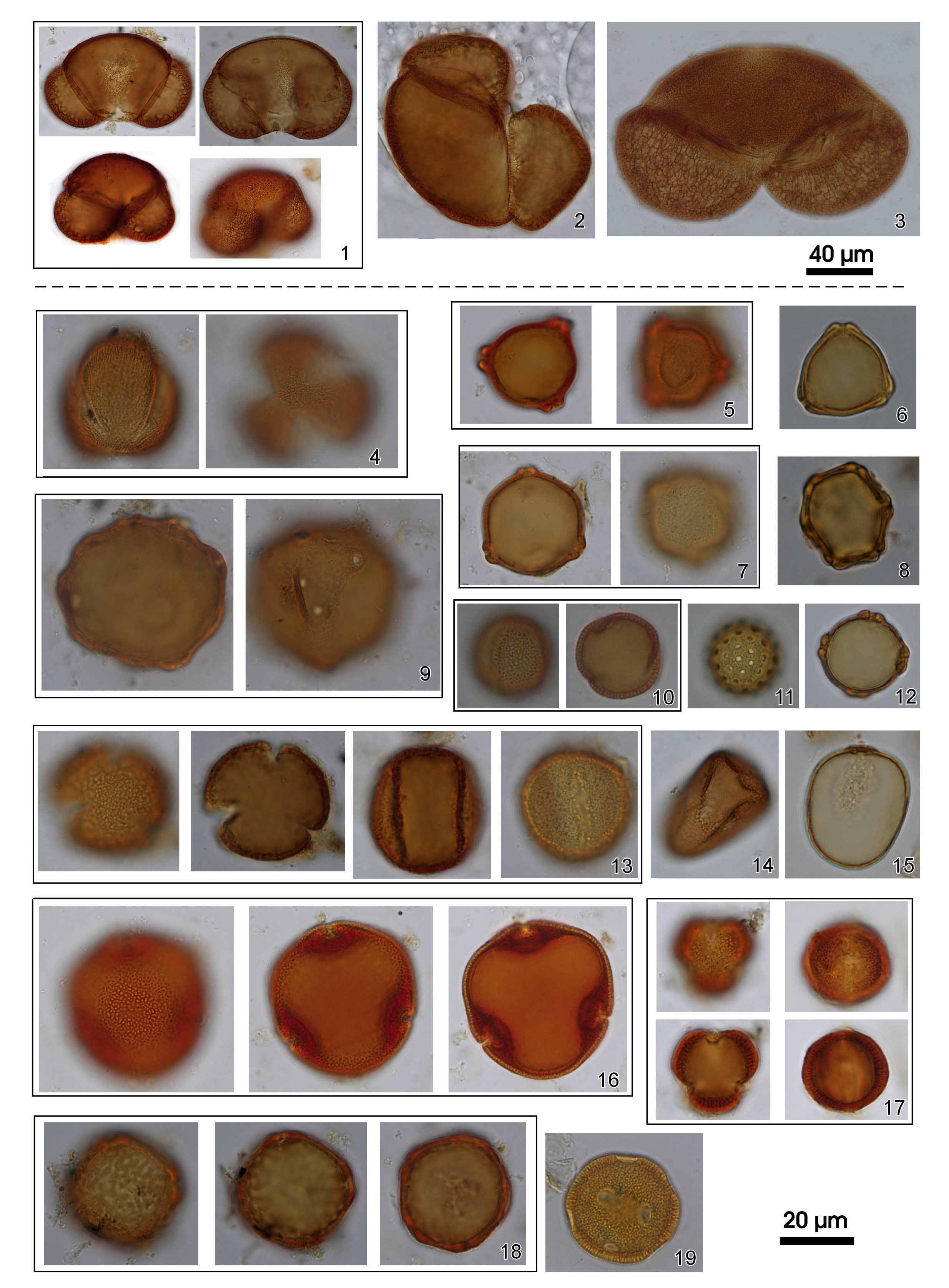


Caption for Pollen plate:

1*.Pinus*, 2. *Abies*, 3. *Picea*, 4. *Acer*, 5. *Betula*, 6.*Corylus*, 7. *Carpinus*, 8. *Alnus*, 9. *Juglans*, 10. *Fraxinus*, 11. Chenopodiaceae, 12. *Myriophyllum*, 13. *Quercus*, 14. Cyperaceae, 15. Poaceae, 16. *Tilia*, 17. *Artemisia*, 18. *Ulmus*, 19. Caryophyllaceae

**Figure S4 Principal component analysis (PCA) of pollen percentage data from Lake Xiaolongwan**

Principal components analysis (PCA) was applied to the terrestrial pollen percentage data to extract the main gradient changes in vegetation. All pollen taxa with relative abundance >2% in at least two samples were used in the analysis. The first and second principal components (PCA F1 and PCA F2) have eigenvalues of 0.71 and 0.15, explaining 71% and 15% of total variance of pollen data respectively (Fig. S3). Broadleaved taxa, *Quercus D,* *Ulmus*, *Juglans*, *Fraxinus* and *Carpinus* have positive loadings on axis 1, whereas temperate mix deciduous and coniferous forest taxa, *Pinus*, *Abies*, *Betula* and some herb taxa, such as *Artemisia* and Chenopodiaceae, have negative loadings. The PCA F1 loadings represent a temperature gradient from warm (positive) to cold (negative) climate conditions.

**Figure S5** **Pollen concentration diagram of Lake Xiaolongwan for the past 5350 years**

Pollen concentrations of temperate mixed deciduous and coniferous forest taxa (*Pinus*, *Betula*, *Carpinus*, *Quercus D* and *Ulmus*) and herb taxa (*Artemisia*) indicate large changes from 3340 BC to 2005 AD. The concentration of mixed deciduous and coniferous taxa, *Pinus* (2~13 N/g×10^5^), *Betula* (0.5~4 N/g×10^5^), and herb taxa *Artemisia* (1~3 N/g×10^5^) increases, while the concentration of broadleaved deciduous forest taxa, *Quercus D* (10~3 N/g×10^5^), *Carpinus* (5~1 N/g×10^5^), *Fraxinus* (2~1 N/g×10^5^), Juglans (4~2 N/g×10^5^) and *Ulmus* (3~1 N/g×10^5^), decreases. The major pollen shift happened from 2000 to 500 BC: a rise in pollen concentrations of *Pinus* (5~10 N/g×10^5^), *Betula* (1~2 N/g×10^5^) and *Artemisia* (1~2 N/g×10^5^) at the cost of decreases in pollen concentrations of *Quercus D* (35~20 N/g×10^5^) and *Carpinus* (20~5 N/g×10^5^).

There is a series of multicentennial oscillations in pollen concentrations between *Pinus* and *Quercus D*. *Pinus* concentrations reach higher values, while *Quercus D* concentrations drop to a low level around 2700 BC, 2200 BC, 1600 BC, 1200 BC, 900 BC, 600 BC, 300 BC, 200 AD, 700 AD, 1200 AD and 1800 AD. Each interval with lower values of *Quercus D* pollen concentrations (*Pinus* higher values) is about 500 year. The regular 11 anti-phase coupled fluctuations between *Pinus* and *Quercus D* existed throughout the past 5350 years.

**Figure S6 Characteristic of pollen percentages and concentrations of *Pinus* and *Quercus D* periodicities**

(A) Univariate spectral analysis results of the *Pinus* and *Quercus D* percentage and concentration time series over the past 5350 years. The spectra are estimated using the Lomb-Scargle Fourier Transform for unevenly spaced data. OFAC and HIFAC are 2 and 1 for both (A) and (B). It is performed by the Welch-Overlapped-Segment-Averaging procedure with 50% overlapped segments for univariate spectra. A Welch window type is employed to reduce spectral leakage. The univariate spectra are bias-corrected using 1000 Monte-Carlo simulation. The number within the graph indicates that significant periodicity of 500 years are above the 99% for A1, B1 and B2, 95% for A2, confidence level based on the χ2 test. The 6-dB bandwidth, determining the frequency resolution, is both (A and B) 0.744 ky^-1^. See ref.4 for details of the method.

**Figure S7 Time series of *Pinus* and *Quercus D* percentage**

(**A**) The percentage of *Pinus* (A1) and *Qerucus D* (B1) was detrended using polynomial fitting. (**B**) The original and 13 points smoothed residuals of *Pinus* (A2) and *Qerucus D* (B2) percentage.

**Figure S8 A comparison of climate proxy records from core Lake Xiaolongwan with Total Solar Irradiance.**

(**A**) 400-600 year band pass filter of Total Solar Irradiance, (**B)** Residuals of PCA F1, **(C)** Residuals of *Qerucus D* percentages, (**D**) Residuals of *Pinus* percentages.

**Reference**

1 Zhao, D. *Vertical vegetation Zone in Changbai Mountain* (China Forestry Publishing House, Beijing, 1980).

2 Li, J., Wu, B. & Sheng, L. *Jilin vegetation* (Jilin Science Press, Changchun, 2001).

3 Appleby, P. G. *et al.* 210Pb dating by low background gamma counting. *Hydrobiologia* **143**, 21-27, (1986).

4 Schulz, M. & Mudelsee, M. REDFIT: estimating red-noise spectra directly from unevenly spaced paleoclimatic time series. *Comput. Geosci.* **28**, 421-426, (2002).

5 Steinhilber, F. *et al.* 9,400 years of cosmic radiation and solar activity from ice cores and tree rings. *Proc. Natl Acad. Sci.* **109**, 5967-5971, (2012).

6 Stuiver, M. & Braziunas, T. F. Sun, ocean, climate and atmospheric 14CO2 : an evaluation of causal and spectral relationships. *Holocene* **3**, 289-305, (1993).
